# Restoring balance in atopic disorders: insights into type 2 immunity and chronic inflammation

**DOI:** 10.3389/fimmu.2026.1793641

**Published:** 2026-06-05

**Authors:** Eric L. Simpson, Oscar Palomares, Brian S. Kim, Klaus F. Rabe, Yamo Deniz, Antonio Martin, Rahin Ghassemebrahimzadeh, Sonya L. Cyr

**Affiliations:** 1 Department of Dermatology, Oregon Health & Science University, Portland, OR, United States; 2 Department of Biochemistry and Molecular Biology, School of Chemistry, Complutense University of Madrid, Madrid, Spain; 3 Kimberly and Eric J. Waldman Department of Dermatology, Icahn School of Medicine at Mount Sinai, New York, NY, United States; 4 LungenClinic Grosshansdorf (member of the German Center for Lung Research [DZL]), Airway Research Center North (ARCN), Grosshansdorf, Germany; 5 Christian-Albrechts University (member of the German Center for Lung Research [DZL]), Airway Research Center North (ARCN), Kiel, Germany; 6 Regeneron Pharmaceuticals, Tarrytown, NY, United States; 7 Sanofi, Vernier, Switzerland; 8 Sanofi, Reading, United Kingdom

**Keywords:** asthma, atopy, COPD - chronic obstructive pulmonary disease, eczema, eosinophilic esophagitis (EoE), helminths, immunity, type 2

## Abstract

Atopic diseases arise from an immunological imbalance where regulatory mechanisms are unable to preserve or restore homeostasis, leading to chronic inflammatory conditions affecting epithelial organs. This may involve homeostasis, deficient or insufficient regulatory T cells (Tregs) or other aberrant regulatory mechanisms. Type 2 (T2) immunity is a conserved response that evolved to combat large helminth parasites (worms), venoms, and toxins involving both innate and adaptive immune pathways. Many T2 cytokines and alarmins act to recruit and activate innate and adaptive immune cells, and they also lead to mucous production, hyperplasia, and tissue remodeling. These responses were designed to enhance expulsion of parasites, repair the barrier and elicit protective mechanical reflexes such as scratching or coughing. Today, with reduced parasitic exposure serving as an opposing influence on T2 immunity, it is hypothesized that T2 responses may be triggered by low amounts of environmental stimuli in genetically susceptible individuals, leading to unchecked T2 inflammation and atopic diseases at multiple barrier surfaces. This paper reviews the evidence linking host T2 immunity with T2 inflammatory mechanisms in atopic diseases and explores the hypothesis that these diseases may be perpetuated from a central imbalance between Th2 vs. Th1, Th3, and Tregs, influenced by tissue-dependent, local environmental-insult-driven innate cell responses, interconnected by a cycle of self-amplifying cytokine signaling.

## 1 Introduction

Type 2 (T2) immunity is an evolutionarily conserved arm of the immune system that combats parasites (including ectoparasitic and endoparasitic helminths), venoms, and toxins, and promotes tissue repair in response to parasitic damage (1–3). Helminths are parasitic worms that were once ubiquitous and are still present in different regions of the world, with repeated exposure of individuals being common (4). Until relatively recently in our evolution, most humans co-existed with or were at risk of parasitic infections with helminths (5, 6). There is archaeological evidence that helminths have affected humans throughout history, including in regions where parasites are no longer prevalent (7, 8). Empirical evidence suggests that historical pandemics may even have imposed positive selection for immune-related genes, specifically gene variants that enhance inflammatory responses, possibly influencing modern day susceptibility to chronic diseases (9–12).

Helminths can be transmitted to both humans and animals via several different routes, including ingestion, insect bites, or skin penetration (13, 14). As helminths complete their life cycles in the body, and migrate through their hosts organs, including the lungs, intestines, liver, and skin, they can cause considerable tissue damage and trigger tissue repair mechanisms (3, 13). The immune system developed to deploy both innate and adaptive T2 responses to kill (cytotoxic effector cells) or expel parasites (neuroimmune mechanisms) and trigger wound repair (barrier homeostasis) (3, 15), whereas parasites, such as helminths, are evolutionarily designed to sustain their presence by suppressing immune responses and avoid killing their host (16). A wide range of immunomodulatory factors have been identified that are secreted by helminths that have the effect of counteracting host immune responses (17). Examples of immune suppression include a study in children with helminth infections reported that a significant reduction in worm burden over a 12-month period in helminth-infected children increased the risk of allergen skin sensitization (18–20), suggesting low-grade parasitic infections dampen allergic mechanisms. Thus, reduced helminth exposure may be one explanation for the enhanced T2 inflammation responsible for atopic diseases. It has been hypothesized that the evolution of T2 diseases could have originated from the natural selection favoring genes that confer an exaggerated T2 immune response as a result of helminth infections, which may clinically manifest as T2 inflammatory diseases (21) in the modern era of low parasitic burden, particularly in urbanized areas.

## 2 The role of T2 immunity in fighting large invading pathogens (e.g., helminths)

**Figure 1:**
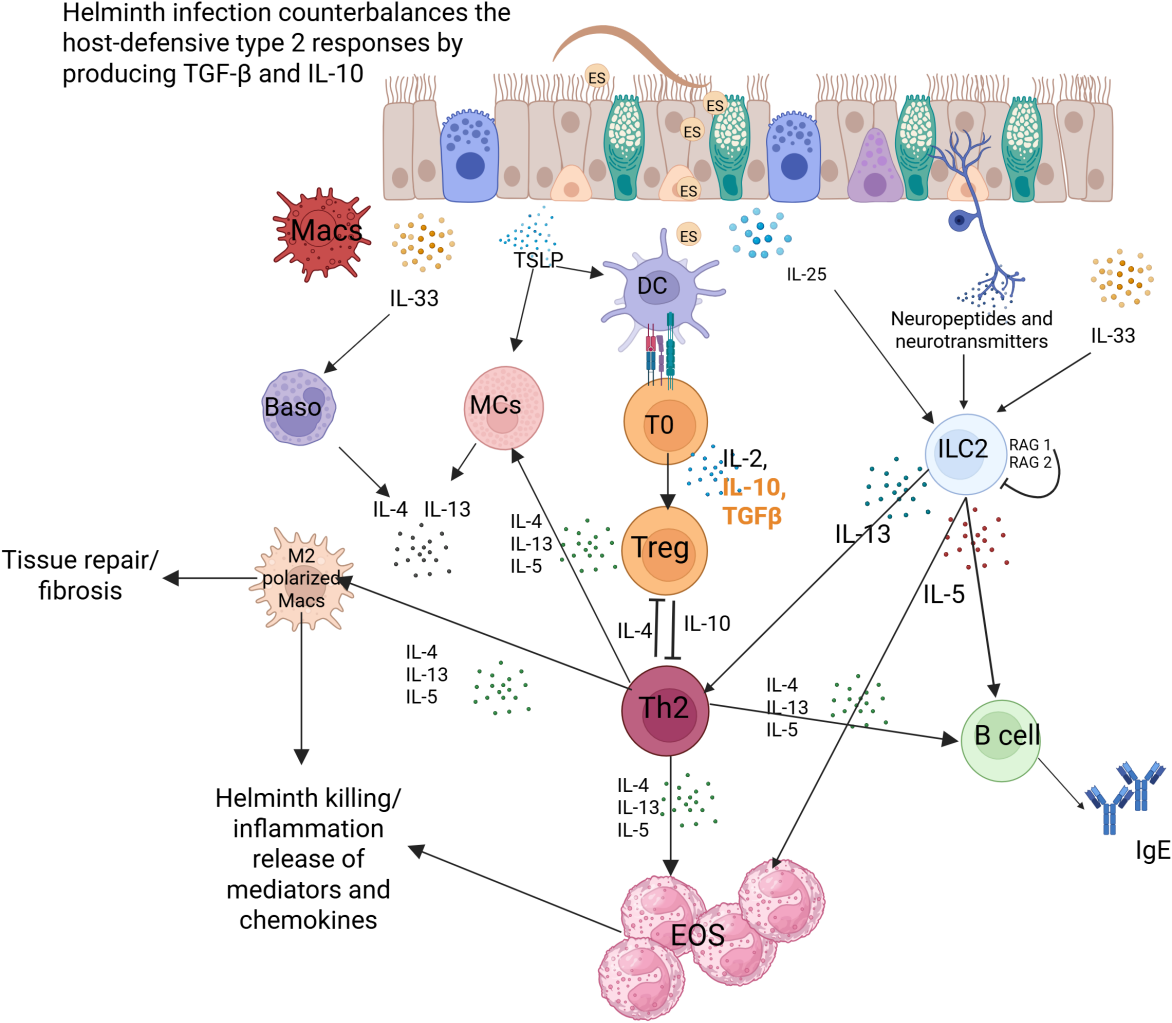
Role of innate immunity in triggering expulsion of helminths and the role of helminths in dampening the immune response through reshaping of the cytokine milieu (3, 23–25). Lung epithelium shown for illustration; similar principles apply to skin and gastrointestinal epithelial surfaces. DC, dendritic cells; EOS, eosinophils; ES, excretory/secretory products; ILC2, group 2 innate lymphoid cell; IL, interleukin; MC, mast cell; NP, neuropeptide; NT, neurotransmitter; RAG, recombination activating gene protein; TGF-β, transforming growth factor beta; Tregs, regulatory T cells; TSLP, thymic stromal lymphopoietin. Adapted from Peng et al. (23). Copyright ^©^ 2022 Peng, Federman, Hernandez, and Siracusa. Adapted from an open-access article distributed under the terms of the Creative Commons Attribution License (CC BY). The use, distribution, or reproduction in other forums is permitted, provided the original author(s) and the copyright owner(s) are credited and that the original publication is cited, in accordance with accepted academic practice. No use, distribution, or reproduction is permitted that does not comply with these terms. Created in BioRender. Hodgkinson, J (2026). https://BioRender.com/anyhvrp.

T2 immunity evolved to fight large parasites, by killing them, and/or by their physical expulsion, which leads to parasite barrier destruction and collateral epithelial damage and repair, as well as host barrier dysfunction in chronic infection (3, 22). A strong T-helper 2 (Th2)-skewed memory T-cell response is induced by helminth infection, which begins at the epithelial surface and is associated with interleukin (IL)-4, IL-5, IL-13, and IL-31, as well as mastocytosis, eosinophilia, and antibody class-switching in B cells producing IgE (3, 23–25). Alarmin cytokines (thymic stromal lymphopoietin [TSLP], IL-25, and IL-33) activate tissue-resident immune cells (dendritic cells [DCs], group 2 innate lymphoid cells [ILC2s], and mast cells), a process which further recruits circulating granulocytes, eosinophils, and basophils, to kill, expel parasitic helminths and toxins, and to repair damage caused to the epithelial barrier (**Figure 1**) (3, 23–26).

Antibody class-switching in B cells producing antigen-specific IgE plays a critical part in linking memory recognition of helminth antigens with rapid, innate granulocytic response and activation of mast cells (MCs) upon re-exposure (**Figure 1**) (23). For example, helminth antigen-bound IgE enables eosinophil recognition of the Fc region of IgE, leading to a granulocytic response in eosinophils to destroy helminths (27, 28).

For most physiological processes of IL-4 and IL-13 intended for host defense, there is a pathological correlate. IL-13 secretion leads to goblet cell hyperplasia and increased mucus production, intended for parasite expulsion, for example via the cough and sneeze reflexes (26, 29). However, these processes cause morbidity in asthma. IL-4 and IL-13 driven epidermal recruitment of cytotoxic effector cells, such as eosinophils to kill parasites by disrupting their membrane lipid bilayer. However, in inflammatory states, eosinophils cause significant damage to the host keratinocytes (30) observed in lung or esophagus tissue remodeling or skin lesions, via a process involving apoptosis of keratinocytes leading to spongiosis (31). IL-4 and IL-13 also promote sensory neuron sensitization, thereby increasing sensory awareness, and urgency to physically expulse a parasite, e.g., via scratching (26).

IL-4 and IL-13 skew macrophages toward a tissue repair phenotype characterized by the expression of factors such as the enzyme arginase-1, the chitinase-like protein Ym1, and resistin-like molecule alpha (Relm-α) (32). This profibrotic healing response via IL-4 and IL-13 has been linked to the development of fibrosis in the lungs of patients with chronic obstructive pulmonary disease (COPD) and asthma (33), and in the information of skin lesions in atopic dermatitis (AD) and prurigo nodularis (PN) (34, 35).

ILC2s produce IL-4, IL-13, and IL-5, acetylcholine, IL-9, methionine-enkephalin peptides, OX-40L, and amphiregulin, which act across multiple organs, activating eosinophils and inducing mucus secreting goblet cell hyperplasia designed to kill and expel helminths (36–46). Epithelial or epidermal tissue damage triggers alarmin production following invasion which in turn stimulates ILC2s, found in high numbers in barrier surfaces and recognized as the innate counterparts of Th cells (47). Alarmins may also directly trigger neurons, where specialized itch-inducing MrgprA3 neurons have been shown to induce IL-33 production in myeloid cells, and in doing so, trigger an immune response to helminths (48). Following the release of neurotransmitters and neuropeptides produced by neurons at barrier surfaces and IL-25 stimulation ILC2s transform from their resting state to inflammatory, circulating ILC2s (37). Neuropeptides such as NMU and CGRP enhance the production of mucus and smooth muscle contraction, critical for physically expelling helminths from the gastrointestinal or respiratory tracts (37, 49). NMU strongly activates ILC2s via the NMUR1 receptor, enhancing the production of T2 cytokines (IL-5 and IL-13). ILC2s lack the receptors resulting from activation of recombination-activating gene endonucleases RAG1 and RAG2 that Th2 cells have, important for antigen specificity, and therefore respond to non-specific alarmin triggers (37, 47, 50). They are nonetheless implicated in the development of AD and other atopic T2 immunity driven diseases. In a murine model of AD, depletion of ILC2s has an additional benefit following depletion of adaptive T cells, they have non-redundant functions in anti-helminth immunity (37, 50–52). These findings suggest that ILC2’s may contribute to the pathogenesis of atopic diseases, independent of adaptive immunity (52). Indeed, ILC2s can transform from their resting state to inflammatory, circulating ILC2s in response to helminth infection and IL-25 stimulation (50, 53). This ILC2 protective response parallels antigen-driven responses, providing an extra innate immune response against infection. Moreover, a phenomenon coined “remote priming” was identified, where local skin inflammation, via IL-33, and ILC2, can allow for remote priming of humoral immunity to bystander antigens in the gut, i.e. leading to the immune system being trained to recognize antigens in the gut following skin exposure (54). When the antigen enters the gut at a later time, the humoral immune system responds, a mechanism potentially leading to food allergy (FA) development in the absence of helminth infection. Together, these observations suggest that across epithelial organs, aberrantly activated memory Th2, ILC2, alarmins, and neuropeptides are potential sources of unchecked inflammation leading to the chronic pathology associated with diseases of the atopic diathesis (55, 56).

## 3 Helminths evolved to establish chronic infection by promoting functional downregulation of host T2 immunity

**Figure 2:**
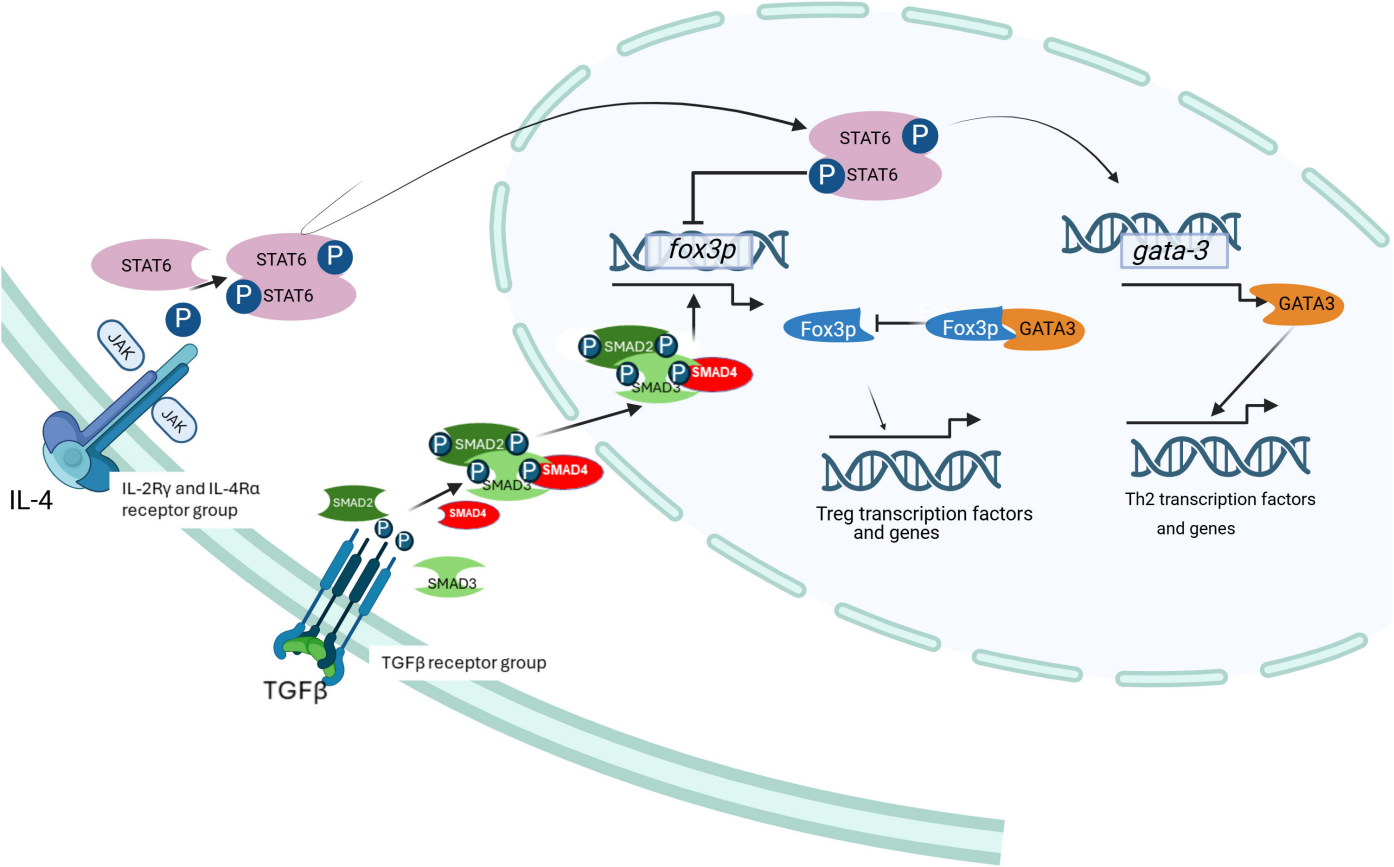
Dampening the T2 response at the transcription factor level is determined by the cytokine milieu (25). IL, interleukin; JAK, Janus kinase; Foxp3, scurfin; SMAD, mothers against decapentaplegic homolog; STAT, signal transducer, and activator of transcription; T2, Type 2; Th, Type 2 helper; TGF-β, transforming growth factor beta; Treg, regulatory T-cell. Created in BioRender. Hodgkinson, J (2025). https://BioRender.com/lg76430.

Innate and adaptive responses to helminths differ with regard to antigen specificity, although both act to release cytokines necessary for helminth destruction and expulsion (57). During the development of adaptive immune responses to pathogens, self-antigens, or environmental allergens, presentation of antigens by DCs to naïve cluster of differentiation (CD)4^+^ T cells causes their differentiation into subsets of effector and memory cells, including Th1, Th2, and Th17 cells, or Tregs. The differentiation into these subsets is controlled by the cytokine environment influencing specific transcription factors (47, 58) (**Figure 2**). Overall, the activity of the effector cells (‘the accelerators’) is limited by Tregs (‘the brakes’), whose differentiation, and maintenance are dependent on the transcription factor Foxp3. Helminth manipulation of Tregs and subsequent downregulation of T2 immunity plays a key role in the parasite’s ability to establish chronic infections (26).

In the presence of helminths, DCs may induce Tregs that produce suppressive cytokines (IL‐10 and TGF‐β). In humans, these act on B cells to switch to IgG4 instead of IgE production, on macrophages to acquire an anti‐inflammatory (M2-like) phenotype, and on effector Th2 cells to block activation and develop a state of tolerance. These pathways inhibit helminth‐specific reactivity, thereby creating a profound anti‐immunity effect (**Figure 2**) (23). In addition, helminths also act to induce the proliferation of myeloid-derived suppressor cells (MDSC), which can act to suppress both innate and adaptive cells, including natural killer (NK) cells and B cells (59).

In addition to influencing cytokine signals, a large number of helminth-associated immunomodulatory molecules have been identified, including proteins, lipids, carbohydrates, neuropeptides, and nucleic acids, which act to manipulate the host immune system (60–62). Some of these molecules are homologous to host molecules, allowing the parasite to manipulate immune cell function by mimicking host proteins, or producing miRNAs that target host gene expression (63).

## 4 Missing “brakes”; how unchecked T2 immunity and can become T2 inflammation

A triggered T2 immunity (by genetic predisposition, or by environmental factors) may become unchecked T2 inflammation without an ability to stop the T2 cytokine driven positive feedback loop implicating several cell types such as Th2, ILC2, neuronal, epithelial, and B cells. Helminth infections may be one of the factors that have led to the selection of immune response genes that provide protection by generating immune signals that override the parasites’ attempts to suppress the immune system. This overriding capacity may also translate into susceptibility to T2 inflammatory diseases in the absence of helminth-mediated downregulation (**Figure 2**), as described above (3, 4). Additionally, the role of ILC2 innate immune cells in regulation of T2 immunity has more recently been uncovered (13, 53, 57, 64).

To facilitate survival when helminth infections were ubiquitous, the host had an evolutionary advantage to counter efforts by helminths to downregulate T2 immunity. An imbalance favoring T2 immunity occurs in the presence of chronic sustained signaling via the IL-4R signal transducer and activator of the transcription 6 (STAT6) axis, disrupting tolerance by facilitating the complete subversion of Treg cells into Th2 cell-like cells (65). The intracellular signaling triggered by IL-4 via STAT6 promotes the transcription of GATA3, directing differentiation toward a Th2 phenotype, producing T2 cytokines, with IL-4 promoting further activation and proliferation of Th2 cells. Furthermore, the transcription factor for Th2 (GATA3) blocks the transcription factor for Tregs (Foxp3), as shown in **Figure 2**. In contrast, Foxp3+ Tregs can reciprocally block GATA3 (and Th2 cell development), but paradoxically, Foxp3+ Tregs also suppress IL-2 production (25) (**Box 1**), a signal which is critical for their own survival and maintenance. This consequently creates an imbalance and forces Tregs to rely on other sources of IL-2, coming from conventional T cells, which may be in short supply in a state of chronic T2 inflammation. Th2 cells typically stop producing significant amounts of IL-2, once they fully differentiate into effector Th2 cells (67); this happens relatively early in the differentiation process, as the presence of polarizing cytokines like IL-4 and IL-5 actively suppress IL-2 production and promote the expression of Th2-specific cytokines like IL-4, IL-5, and IL-13 instead (23). Therefore, given that the amount, and kinetics of the cytokine milieu determines the balance between Th2 and Tregs, the chronic autocrine presence of IL-4 and other T2 cytokines may perpetuate Th2 and reduce Treg cells development, in the absence of an extraneous (e.g. helminth parasite) source of IL-10 and TGF-β (3, 23, 25).

BOX 1 Naïve CD4 T cells initially produce IL-2 upon activation, which is necessary for their proliferation and differentiation. Conventional T cells are the main source of IL-2. IL-2 is a T-cell growth factor essential for the proliferation of T cells and the generation of effector and memory cells, and it is also essential for the generation, survival, and functional activity of Treg cells. Thus, IL-2 has dual and opposing functions in balancing T-cell populations: maintaining Treg cells to control immune responses and stimulating conventional T cells to promote immune responses (25, 66). In addition, Tregs express high levels of CD25, the receptor for IL-2, which enables sequestration of IL-2 and inhibits T effector cell activation, as a suppressive mechanism.

Tregs are one of the key cells promoting the maintenance of immunological tolerance, and clinical evidence for the imbalance favoring Th2 over Tregs exists. It has been demonstrated that a high serum level of IL-4 decreased the number of Tregs in patients with severe AD (68). Naturally occurring mutations in the *FOXP3* gene in humans and mice cause immunopathology that has a major Th2 component (69–71). Humans develop the IPEX syndrome as a result of loss-of-function mutations in *FOXP3* (69). Clinical manifestations of IPEX (also called X-linked autoimmunity-allergic dysregulation syndrome or XLAAD) include autoimmunity together with severe atopy, eczema, food allergies, and eosinophilic inflammation (70). Low-dose IL-2 has been shown to partially rescue Treg function in some autoimmune diseases (72). Recently, a Treg-selective IL-2 receptor agonist (rezpegaldesleukin) has demonstrated efficacy in the treatment of AD, in early phase clinical trial, and showed a sustained increase in Tregs, underscoring the crucial role of Tregs (24, 73) in controlling inflammatory responses. In addition to Tregs, ILC2 cells may also form part of the immune response in T2 inflammatory disorders.

Recent findings suggest the expression of RAG endonucleases expression may serve as a regulatory mechanism for T2 activation/suppression not only in T or B lymphocytes, but also in ILC2 (47), despite the absence of V(D)J recombination promoting the assembly of the antigen-specific receptors seen on T and B cells. RAG-deficient genetic human diseases, like Omenn syndrome (OS) are characterized by T2 inflammatory pathology including severe eczema, IgE, and eosinophilia. Patients with OS carry mutations of *RAG1* or *RAG2,* resulting in severe combined immunodeficiency with absence of mature T and B cells. In addition, RAG expression has been suggested to imprint suppressed proliferative and T2 inflammatory functions on ILC2s in the setting of AD-like disease in mouse models (47). The mechanisms underlying the propensity of T cells with hypomorphic RAG activity to preferentially develop into the Th2 subtype are unclear (47). Prior studies have found a role for Treg cells in controlling T2 skewing of transferred T cells in RAG-deficient hosts (74). Additionally, increased T2 cytokine production from RAG-deficient ILC2s may enhance expansion of the oligoclonal Th2 cell populations, IgE induction, and eosinophilia observed in RAG-deficient states like OS (74, 75). Moreover, RAG expression modulates the function of innate NK cells. Murine NK cell deficiency was associated with enhanced T2 inflammation in the skin, suggesting that NK cells play a critical immunoregulatory role (76). Lymphoid acquisition of RAG activity may represent a newer evolutionary mechanism that regulates ancient innate immune cell programs in addition to enabling development of newer antigen-specific adaptive immune cell populations.

Interestingly, OX40L expression by ILC2s is required for IL-33-driven Th2 and Treg cell expansion, indicative of a critical role for these innate cells towards both inflammation and regulation (46). Downstream direction towards either inflammation or regulation may therefore rely on the T2/regulatory cytokine milieu.

## 5 Mechanisms of T2 inflammation reminiscent of defense mechanisms

T2 inflammatory diseases share many common elements, including the interplay between the immune response and the altered epithelial barrier function (77, 78). The epithelia of the lungs, nose, skin, and gastrointestinal tract have differing, complex, and highly dynamic processes, with continuous self-renewal, and repair mechanisms following damage. Breaking the vicious cycle of epithelial disruption and inflammation allows the epithelium to heal and restore its natural barrier function, as well as reducing inflammatory processes that sustain chronic disease processes (79).

**Figure 3:**
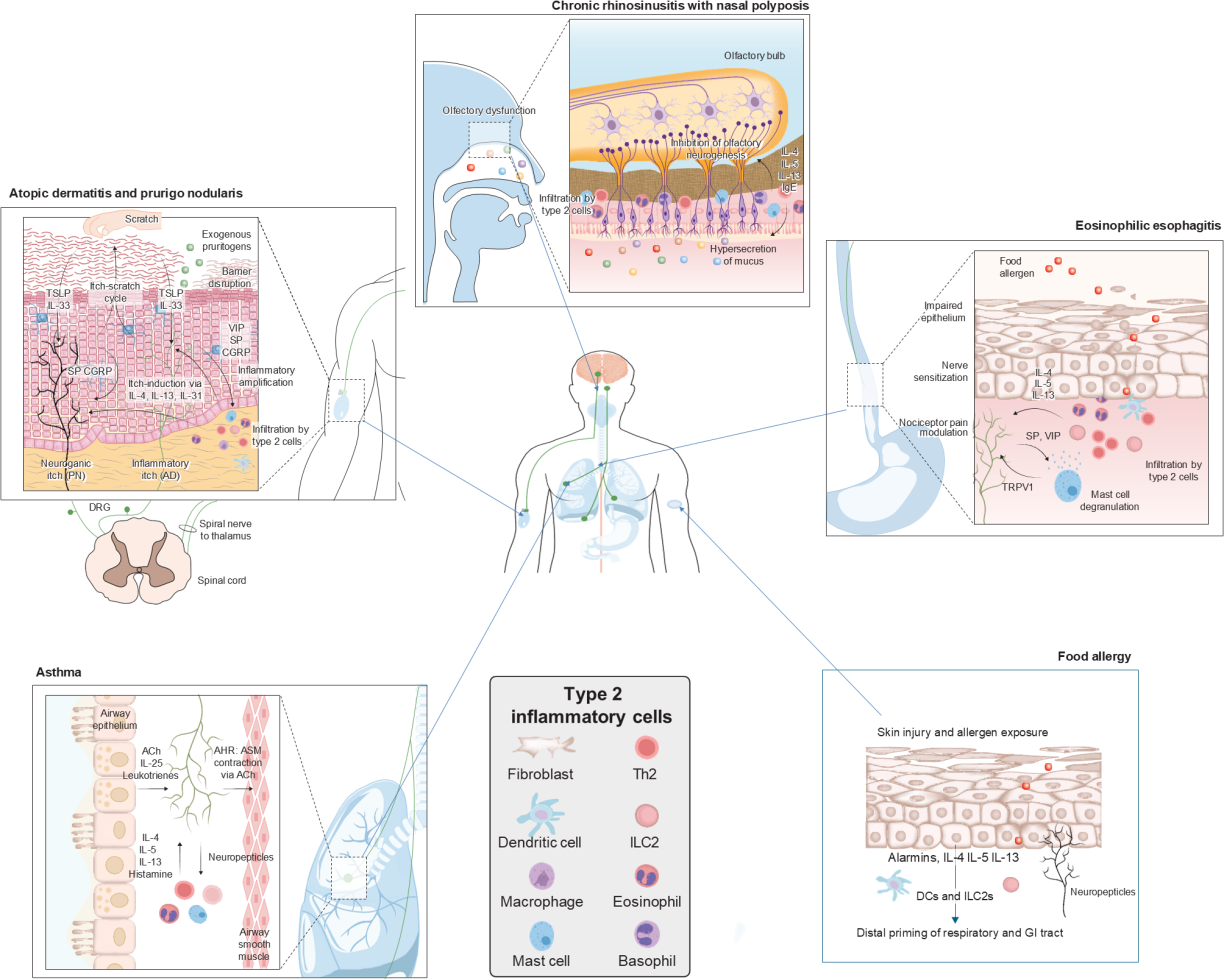
Neuroimmune interplay in conditions associated with Type 2 inflammatory diseases (15, 34, 82). IL, interleukin; Th, Type 2 helper; TSLP, thymic stromal lymphopoietin. Adapted from Kim et al. (15). Copyright: ^©^ 2023 The Authors. Published by Elsevier Inc. on behalf of the American Academy of Allergy, Asthma & Immunology. Adapted from an open-access article distributed under the terms of the Creative Commons Attribution License (CC BY). The use, distribution, or reproduction in other forums is permitted, provided the original author(s) and the copyright owner(s) are credited and that the original publication is cited, in accordance with accepted academic practice. No use, distribution, or reproduction is permitted that does not comply with these terms.

Aberrant T2 responses are present in AD, asthma, allergic rhinitis, and sometimes in IgE mediated FA, as well as chronic rhinosinusitis with nasal polyps (CRSwNP), eosinophilic esophagitis (EoE), PN, eosinophilic gastrointestinal diseases (EGIDs): and in some cases of COPD, and inflammatory bowel diseases (IBD) such as ulcerative colitis (15, 34, 79–82) (**Figure 3**).

Evidence across the licensed biologic therapies for T2 diseases, and knowledge of disease pathologies, indicate a key role of IL-4 in multiple T2 inflammatory diseases across multiple body systems/organs, together with the roles of IL-13, IL-5, IL-31, IgE, and TSLP across different T2 inflammatory diseases (79, 83).

### 5.1 Barrier disruption

Alarmins and a chronic presence of T2 cytokines at the barrier act to promote ILC2 activation and Th2 cell differentiation, polarization, and proliferation, as well as B cell isotype switching, maintaining B cell clones as well as causing a range of adverse effects, such as immune cell trafficking, tissue remodeling, and microbiome imbalance, culminating in epithelial barrier disruption (4, 79, 84). The barrier disruption in AD has been reviewed elsewhere (34). Briefly, T2 inflammation leads to the inhibition of epidermal protein expression, including FLG, loricrin, and involucrin, and the production of short-chain fatty acids instead of preferred longer chains, ceramides, and non-hydroxy fatty acids (85, 86). This has been demonstrated in both non-lesional and lesional skin, indicating that patients with AD are vulnerable to environmental allergens and pollutants before the appearance of AD lesions. In areas of active AD, eosinophils, basophils, and MCs release histamine and major basic protein that can worsen inflammation as well as skin barrier disruption by downregulating the structural proteins in the stratum corneum and disruption of tight junction proteins. Similarly, although data is more limited, it has been demonstrated that the bronchial epithelial barrier of the lungs in patients with asthma is compromised. In the lungs, environmental toxins and allergens can damage the epithelial barrier, triggering an inflammatory response which can result in airway hyperresponsiveness and tissue remodeling (87). Bronchial biopsies from asthmatic subjects displayed disruption of tight junction proteins (88). In the gastrointestinal tract, infections, changes in diet, sensitization to foods, and toxins can lead to a damaged epithelial barriers that allows allergens to reach the submucosa, which leads to a T2 inflammatory response (89). The damage caused to the epithelium can lead to colonization by opportunistic pathogens, further driving an inflammatory response (90). Together, these pathogenic phenomena mirror the mechanisms of defense against helminth cuticle/tegument, consisting of structural proteins such as collagen, keratin-like molecules, and glycoproteins, as well as lipid layers (91).

### 5.2 Neuroimmune dysfunction

Neuroimmune responses in T2 inflammation involve bidirectional interactions between sensory neurons and T2 inflammatory mediators, resulting in sensitization (15). Such neuroimmune changes are characterized in AD, PN, CRSwNP, and asthma, but less so in EoE (15, 35). Moreover, as noted earlier, in a murine model, skin inflammation can lead to remote priming via ILC2. Distal effects in the gut via neuropeptides and neuroimmune mechanisms mediated via ILC2 have also been noted, underscoring the potential role of neuroinflammation towards the so-called “atopic march” (37, 47).

In Type 2 Inflammatory (T2I) conditions, mechanical reflexes, such as scratching, airway constriction, coughing, sneezing, and lung and gastrointestinal motility, intended to protect barrier surfaces and expulse pathogens, are triggered by direct activation of sensory neurons, often in concert with autonomic input to the target organs, leading to changes and damage to the epithelium and manifesting as a range of conditions (**Figure 3**) (15, 34, 82). AD is characterized by itchy skin lesions and increased risk of skin infections, whereas patients with PN have intensely pruritic, hyperkeratotic nodules, both result in neuronal remodeling (15). In AD, decreased innervation in the epidermis is observed. This reduction may represent nerve damage to mechanoreceptors from chronic rubbing or scratching for example, or chronic activation of pruritogens, with IL-4Rα blockade in humans, nerve length and branching has been shown to be restored using imaging techniques in skin biopsies (92). T2 asthma is characterized by inflamed and narrowed airways, and airway hyperresponsiveness (AHR), leading to loss of expiration volume and cough, tissue remodeling and an increased sensitivity of neurons (15). CRSwNP manifests as nasal polyps and loss of sense of smell (15). EoE leads to dysphagia, with esophageal furrowing, and erosion. Other eosinophilic gastrointestinal conditions (collectively known as EGIDs) which are associated with a range of symptoms consistent with dysregulated neural control, depending on the tract wall layer involved (15, 93).

## 6 Maintenance of T2 inflammation is facilitated by secondary lymphoid organs and sometimes by tertiary lymphoid structures

Emerging evidence implicates the formation of tertiary lymphoid structures (TLS) in some atopic conditions through T2I-mediated lymphoid neogenesis (94). TLS (also referred to as ectopic lymphoid tissues) are non-encapsulated clusters of lymphoid cells that form as a result of chronic inflammation within non-lymphoid organs and have similar characteristics and functions to lymph nodes (secondary lymphoid organs [SLO]) but lack a protective membrane (1). In contrast to their possible protective role in lung infections, TLSs may have pathogenic roles in chronic inflammatory lung diseases such as asthma and COPD (1).

SLO have evolved to support the production and maintenance of tissue-resident memory cells, including Th2 cells and IgE-producing B cells, as well as the development of effector cells when exposed to allergens (26),. TLS develop in chronic conditions, and create dynamic immunological niches, promoting adaptive immunity (and unchecked immunity in the case of T2I for example) by direct exposure to diverse stimuli from inflamed environments, due to their unencapsulated structure, which makes them distinct from SLOs (26). As a result, they may also facilitate local morbidity and atopic comorbidity development/progression.

TLS have evolved to occasionally have separate T and B cell compartments (although they may be intermingled), with germinal centers containing follicular dendritic cells, and high endothelial venules, and act as reservoirs for memory B cells. IL-4 and IL-13 play a role in maintaining memory and expanding effector T and B cells, causing rapid and persistent inflammatory markers sustaining the disease state (95). In inflammatory conditions the presence of TLS seem to be unfavorable (95).

## 7 Restoring control: treatment strategies in asthma, COPD, CRSwNP, AD, and EoE

### 7.1 T2 asthma

In T2 asthma, inflamed and narrowed airways and AHR lead to loss of expiration volume and cough, as shown in **Figure 3** (96). Enhanced bronchial smooth muscle contraction is one of the causes of AHR. AHR correlates with the severity of asthma and with the level of treatment needed to control symptoms (96). The production of IgE, dependent on IL-4 and IL-13, is linked to respiratory symptoms, lung function, infections, airway remodeling, and bronchial hyperreactivity (97, 98) In some patients, eosinophil differentiation is increased, promoted by IL-5, and TLS may form as a result of antigens/self-antigens. In the lung, TLS, commonly referred to as isolated aggregations of lymphoid cells, and have been found in 70-100% of patients with airflow obstruction (99). Anti-IgE therapy has been studied using omalizumab, but it is not efficacious in all patient populations, especially among adults with severe asthma with older age onset, perhaps indicating that IgE contributes to a greater extent to pathophysiology in younger patients, and those with allergic asthma, prior to remodeling impacting tissue function (100). Inhibitors of Bruton’s tyrosine kinase (BTK), which act to inhibit the FcϵRI pathway and mediate IgE signaling, have been used for the treatment of B cell malignancies and are currently undergoing evaluation in T2I asthma.a (101, 102). Of note, an IL-4Ra-targeting biologic (dupilumab) and BTK inhibitor (remibrutinib) have been approved in 2025 for the treatment of chronic spontaneous urticaria in the United States (103–105). Inhibition of Janus kinase (JAK) receptors is also being investigated both as inhaled and oral therapies, due to their downstream inhibition of Type 2 signaling (106). Of the therapeutic agents targeting inflammation markers tested for T2 asthma, key targets include IL-4Rα, IL-5, IL-5Rα, IL-33, IL-33R, JAK, IgE, BTK, and TSLP.

### 7.2 T2 chronic obstructive pulmonary disease

Allergens, inhaled particles, cigarette smoke, and self-antigens can initiate the formation of TLS in the lung. While TLS have a protective role in lung infections, they have pathogenic roles in chronic inflammatory lung diseases such as asthma and COPD (33, 107).

The presence of elevated Type-2 inflammatory biomarkers is associated with greater lung function loss, even among participants with no identifiable obstructive lung disease at baseline (108). In COPD, 20–40% of patients have T2 inflammation, and this has been associated with a higher risk of exacerbation, and increased B cell numbers are present in the damaged lung tissue (109–111).

The presence of TLS in COPD is associated with disease severity, and TLS increase in numbers as a patient ages (1). Additionally, they contribute to the chronic inflammatory processes that characterize COPD, leading to tissue damage and progression of the disease (82).

Current therapies used for T2 COPD target IL-4Rα, and IL-5 (112, 113). Biological therapeutic targets being studied for COPD include IL-5, IL-5Rα, IL-33, IL-33R, Suppression of Tumorigenicity 2 (ST2), and TSLP.

### 7.3 Chronic rhinosinusitis with nasal polyps

Olfactory neuron dysfunction in CRSwNP contributes to sensory dysfunction (84, 114). Recent studies point to a role for B cells in the development of CRSwNP, and TLS have been identified in the nasal polyps (NPs) found in CRSwNP in some, but not all studies (115–117). Current biological therapies approved for CRSwNP include those targeting IL-4Rα, IgE, and TSLP.

### 7.4 Atopic dermatitis

In patients with AD, ILC2 cells, Th2 cells, and B cells are found at higher levels in the skin and blood, and they contribute to ongoing inflammation. Memory tissue-resident B cells have been identified in AD, and B cell depletion therapies have been shown to reduce inflammation in severe AD (97, 118, 119). Furthermore, NK cells are “boosted” by immunotherapy with an IL-15 super agonist in a murine AD model, and symptoms are improved (76). Additionally, TLS are postulated to be present in AD, as they have been found in other immune-mediated skin diseases, such as pemphigus vulgaris and the skin manifestations of systemic lupus erythematosus and hidradenitis suppurativa (118–121).

One of the pathogenic mechanisms in AD is barrier disruption, with IgE possibly contributing to AD flares in some patients, however controlling IgE alone does not provide meaningful clinical improvement, apart from in children with severe AD where therapy with omalizumab has demonstrated moderate benefit (82, 122). Current approved therapies for AD target IL-4Rα, JAK1/2, IL-13, IL-31, phosphodiesterase-4, aryl hydrocarbon receptor (123, 124). Therapeutic targets currently being investigated include IL-2, OX40/OX40L, BTK, IL-2 inducible T-cell kinase (ITK), STAT6 and IL-33 (123, 125, 126).

Of note, other dermatologic diseases (e.g. prurigo nodularis) are effectively treated by targeting type 2 inflammation markers (127).

### 7.5 Eosinophilic esophagitis

The impaired barrier function of the esophageal mucosa in patients with EoE allows food and ingested antigens to penetrate, promoting chronic eosinophil-predominant inflammation (128). TSLP and IL-33 cause Th2 cells to release IL-4, IL-5, and IL-13. The presence of IL-4 and IL-13 induces dilated intracellular spaces and basal cell hyperplasia in the esophageal epithelia, whereas IL-5 and eotaxin-3 promote infiltration by eosinophils and possibly by MCs and basophils, and increase B cells and subsequent IgE production (128).

In the gastrointestinal tract, T2 immunity acts to counteract ingested pathogens that can cause dysbiosis and diarrhea. Interestingly, the clinical role of IgE in EoE is still unclear but may be linked to food sensitization (15, 129). Although EoE can be triggered by food allergens, dense infiltration by IgG4-positive plasma cells has been observed around the vessels of the lamina propria of adult EoE patients (130). Current therapies for EoE target IL-4Rα, targets in development for EoE and IBD include IL-13, and IL-5Rα (131).

## 8 Implications of targeting the T2I pathway on T-helper cells immune balance – clinical observations of paradoxical reactions

Case reports of paradoxical reactions where Th1 or Th17-driven conditions following anti-IL-4Rα treatment for AD have been published, such as psoriatic arthritis, and psoriasis, or where Th2-driven conditions such as AD emerge following anti-IL-17/23 treatment in psoriasis (132–136). Some have attributed these manifestations to a polarization or “switch” of the immune system towards opposing T-helper phenotypes. Case reports of patients that developed psoriasis while being treated for AD with JAK inhibitors suggests that broader blockade of cytokines does not prevent phenotypic switching (137, 138). The effect of IL4Rα blockade on T cells has been studied in circulating skin-homing T cells in patients with AD, following short, and long-term treatment, to evaluate whether dupilumab treatment induces long-lasting T-cell polarization. Dupilumab is a human monoclonal antibody that binds to IL-4Rα and inhibits signaling of the canonical T2 cytokines, IL-4, and IL-13. Dupilumab treatment rapidly inhibited IL4Rα, which was accompanied by an early functional effect specifically on skin-homing T cells without affecting overall Th2 Type cell skewing in the long-term (139). This argues for the possibility that Th1 or Th17 phenotypes become apparent in patients with a non-classical mixed-inflammatory phenotype at baseline, and these are potentially transitory until a homeostatic response from Tregs is achieved. Further the relatively low number of observations of this phenomenon suggests this may occur in patients with clinically relevant T cells at baseline co-expressing lineage-specifying transcription factors for both Th1 and Th2 or Th17 and Th2. Further evidence for this comes from the previously mentioned case reports (58, 137, 138).

## 9 Discussion

**Figure 4:**
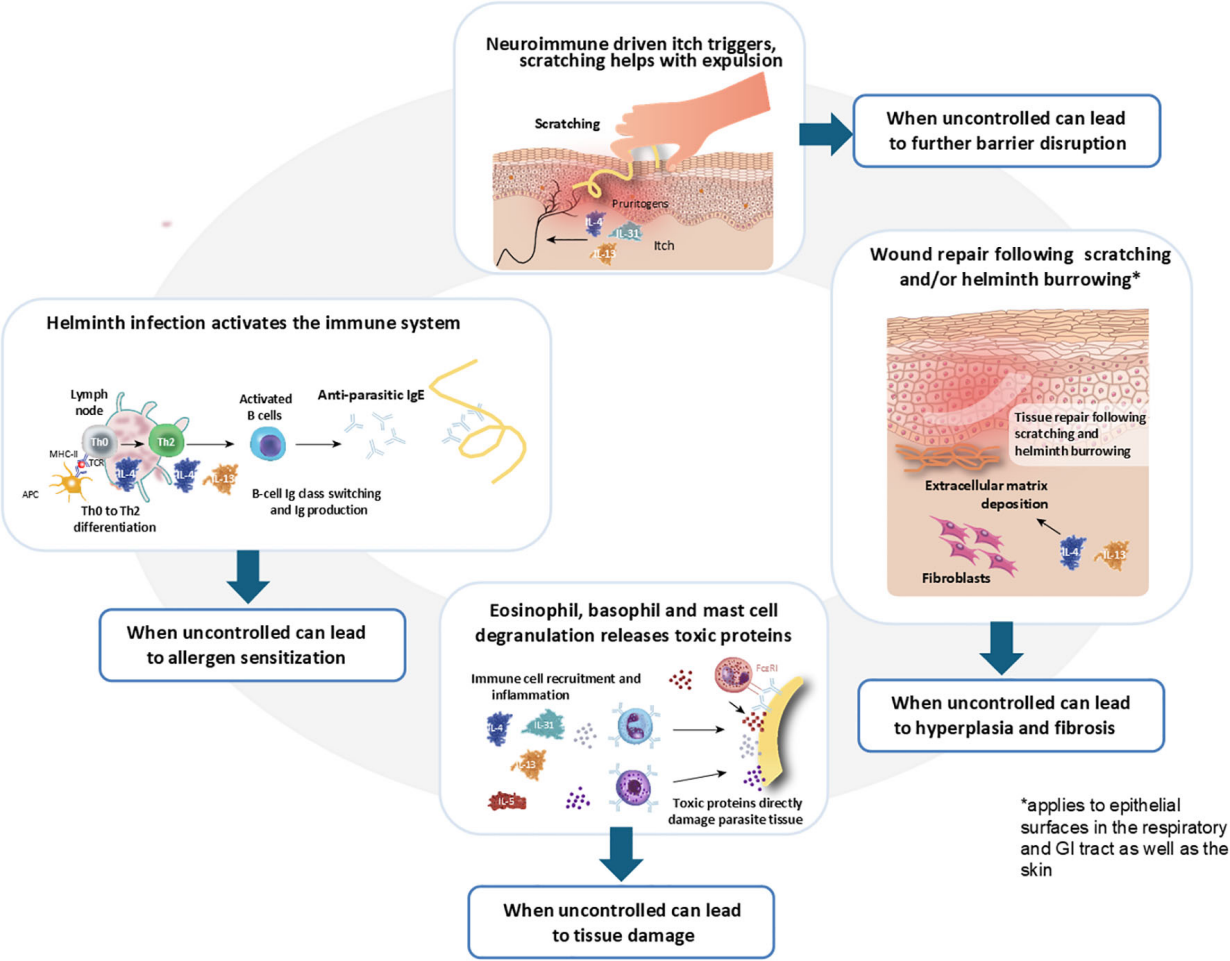
T2 immunity can become T2 inflammation when there is an imbalanced presence of type 2 cytokines (33, 140–142). Epidermis shown for illustration; similar principles apply to respiratory and gastrointestinal epithelial surfaces. APC, antigen presenting cell; IL, interleukin; MH4, major histocompatibility protein Th2, Type 2 helper.

In this review article, we have explored the hypothesis that T2 diseases evolved from unchecked immune responses of the human immune system to combat helminth infections (**Figure 4**). T2 immunity is a carefully orchestrated immune response intended to maintain barrier homeostasis in the various epithelial layers found in skin, respiratory, and gastrointestinal tracts. Helminth downregulation of T2 immunity plays a key role in their ability to establish chronic infections (3, 4). T2 immunity can become T2 inflammation and lead to atopic or T2 inflammatory diseases (in the absence of adequate immune regulation) and may lead to a cascade of additional T2 conditions via the atopic march (21, 107, 143). Thus, hindering the specific chronic signals of T2 inflammation may provide the missing immune regulation in T2 diseases, contributing to restoration of immunological and epithelial structural integrity (21). MCs, ILC2s, eosinophils, basophils, and memory B cells, as well as IgE sensitization, play a critical role in the progression of atopic conditions and comorbidities (15, 144). A strong association has been observed between severe AD and challenge-confirmed IgE-mediated FA in the clinical setting. Among infants attending a dermatology department, 80% of infants with severe AD had probable FA based on high sensitization levels to cow’s milk, egg white, or peanut (145). Although AD in infants and young children can resolve, there is a well-recognized increased risk of sequential progression from AD to other atopic diseases, including FA, allergic rhinitis, allergic asthma, and allergic rhino conjunctivitis (143). The mechanisms underlying the development of AD and subsequent progression to other atopic comorbidities, particularly FA, are not completely understood are the subject of intense investigation (107, 146). However, some evidence exists that IgE sensitization (with or without barrier dysfunction) drives the development of atopic comorbidities, and some experts speculate that without IgE sensitization, there is no atopy or march (147). This process is likely exacerbated in individuals with disrupted barriers (85).

Clinical studies of targeted therapies, such as biologic therapies, have helped to elucidate the mechanism of T2 diseases, and identify the important mechanisms in each specific disease (34, 35, 79, 148–153). T2 immune dysregulation involves many different cell types and inflammatory mediators, and there are differences by epithelial type, evidenced by different therapies being effective in some T2 diseases but ineffective in others (21). To date, dupilumab has demonstrated efficacy, and safety across a range of different epithelial organs where T2 inflammatory diseases are observed, likely attributable to the role of both IL-4 and IL-13 in the perpetuation of not only the Th2 autocrine feedback loop, resulting in the maintenance of chronic Th2 polarization, but also contribution to ILC2 activation at mucosal surfaces, and maintenance of cytokine milieu imbalance hampering Treg development. Given the critical importance of T2 cytokine signaling on overall dysregulated innate cell functions such as M2 macrophage and NK cells, as well as neuronal sensitization, the overall reduction of T2 signaling from both major sources, systemic memory (Th2) and local innate lymphocytes likely explains the cross-organ efficacy of targeting IL-4 and IL13. Clinical experience has shown that IL4Rα blockade has a broader impact on combined T2 inflammatory diseases than IL-5, IL-13, IgE, IL-31, or IL-33 blockade (83, 149, 154). However, it should be noted that some patients do not respond adequately to dupilumab therapy or need additional add-on treatments to achieve results (155). Further research is required to better understand the different disease endotypes and the relative contribution of different inflammatory blockers. While targeting multiple cytokines may seem advantageous, a broader blockade is not necessarily better for every patient or condition. JAK inhibitors inhibit multiple cytokines by targeting the signaling pathway common to multiple cytokine receptors (156). Unlike more precise inhibition of the central drivers of T2 inflammation, JAK inhibitors have been shown to increase susceptibility to some infections (157).

## 10 Conclusions and future perspectives

With less helminth exposure contributing to downregulation, it is hypothesized that T2 responses may be triggered in genetically susceptible individuals and accelerated by Type 2 cytokines common to innate and adaptive cells, leading to unchecked T2 inflammation and atopic diseases at multiple barrier surfaces. We propose that IL4Rα blockade contributes to the downregulation of the common cytokines IL-4 and IL-13 across epithelial organs, perpetuating this conserved arm of the immune system, reducing unchecked T2 inflammation. Recent work has highlighted different disease trajectories in some patients that develop additional T2 comorbidities (158). Although targeting IL-4 and IL-13 pathways has been effective in treating T2 inflammatory conditions throughout the skin, the respiratory tract as well as the upper gastrointestinal tract, it is important to note that disease heterogeneity and differing endotypes may impact treatment responses. Further studies are therefore necessary to deepen our understanding of type 2 inflammatory disease endotypes in the context of local tissues and environmental insults, and to help identify which pathways are most significant for each disease endotype.
