# Supplementary material for: Restoring balance in atopic disorders: insights into type 2 immunity and chronic inflammation
Source: Front Immunol. 2026 Jun 5;17:1793641. doi: 10.3389/fimmu.2026.1793641 (PMC13279686; doi:10.3389/fimmu.2026.1793641)
Supplement: Supplementary file 1 [file SupplementaryFile1.docx]

**Restoring Balance in Atopic Disorders: Insights into Type 2 Immunity and Chronic Inflammation**

Atopic disorders are immune-mediated conditions that occur at barrier surfaces of the body, including the skin, respiratory tract, and gastrointestinal lining—areas where the body interacts with the external environment. These disorders include atopic dermatitis (eczema), asthma, and eosinophilic esophagitis. Factors such as allergens, parasitic worms, and other environmental triggers can affect these surfaces, and studying their impact on the immune system helps us better understand the mechanisms underlying these diseases.

T2 immunity happens when the body produces specialized T2 signals called cytokines, which orchestrates the immune response.

Over time, parasites have developed methods to reduce the immune response directed at them, to continue to live and reproduce in humans. One way by which parasites survive inside the body is by reducing the ability of the immune system to produce T2 cytokines.

In atopic diseases, the immune system is disrupted, leading to chronic T2 inflammation, causing symptoms similar to those intended to fight helminths, such as barrier dysfunction and damage, skin changes such as thickening, and symptoms like itching and lung irritation. The body’s production of cytokines becomes uncontrolled, causing T2 inflammation.

By understanding T2 immunity and its role in chronic inflammation, targeted therapies that block uncontrolled T2 cytokines address the shared root causes of atopic disorders. Therapies like dupilumab, which blocks the action of two cytokines (IL-4 and IL-13), have proven effective in reducing symptoms across various atopic conditions. Such treatment can provide relief, offering hope for individuals with these conditions.
